# Evidence for benefits from treating cervical ectopy: literature review

**DOI:** 10.1590/S1516-31802008000200014

**Published:** 2008-03-06

**Authors:** Luís Carlos Machado, Ana Sílvia Whitaker Dalmaso, Heráclito Barbosa de Carvalho

**Keywords:** Uterine cervical erosion, Uterine cervicitis, Cervical intraepithelial neoplasia, Uterine cervical dysplasia, Uterine cervical neoplasm, Erosão cervical uterina, Cervicite uterina, Neoplasia intra-epitelial cervical, Doenças do colo do útero, Neoplasia do colo do útero

## Abstract

**CONTEXT AND PURPOSE:**

Uterine cervical ectopy (cervical erosion) is today considered to be a physiological condition, but there still seems to be a strong tendency towards treating it. The purpose of this study was to review the medical literature for evidence regarding benefits from treating cervical ectopy.

**METHODS:**

The following databases were reviewed: Medical Literature Analysis and Retrieval System Online (Medline), Excerpta Medica Database (Embase), Literatura Latino-Americana e do Caribe em Ciências da Saúde (Lilacs) and Cochrane Library databases. In addition, six medical textbooks were consulted.

**RESULTS:**

The review showed that: 1) there is probably an association between ectopy and higher risk of *Chlamydia trachomatis*, human papillomavirus and human immunodeficiency virus infection; 4) there is probably an association between ectopy and cervical intraepithelial neoplasia; 5) there is an association between ectopy and mucous discharge and nocturia; and 6) there is no evidence of an association between ectopy and cervical cancer, or of protection against cervical cancer associated with ectopy treatment.

**CONCLUSIONS:**

1) No data were found in the medical literature to support routine treatment for ectopy; 2) Treatment could be recommended for symptom relief, but more symptoms are attributed to ectopy than could be demonstrated in a controlled study; 3) Further studies to test the hypothesis of protection against cervical cancer associated with treatment are necessary.

## RESUMO

**CONTEXTO E OBJETIVO:**

A ectopia do colo do útero é hoje considerada um fenômeno fisiológico, mas parece ainda haver uma forte tendência no sentido da intervenção (tratamento). Este estudo se propõe a realizar revisão da literatura buscando evidências de benefícios conseqüentes ao tratamento da ectopia.

**MÉTODOS:**

Pesquisa nas bases Medical Literature Analysis and Retrieval Sysem Online (Medline), Excerpta Medica Database (Embase), Literatura Latino-Americane e do Caribe em Ciências da Saúde (Lilacs), Biblioteca Cochrane e seis livros especializados.

**RESULTADOS:**

A revisão mostrou que: 1) existe provavelmente associação de ectopia com infecção cervical por *Chlamydia trachomatis,* pelo vírus HPV e maior risco de soroconversão para HIV; 2) existe provavelmente associação entre ectopia e neoplasia intra-epitelial cervical; 3) existe associação com mucorréia e nictúria; 4) não existem evidências sobre associação entre ectopia e câncer de colo do útero nem sobre proteção contra este câncer proporcionada pelo tratamento da ectopia.

**CONCLUSÕES:**

1) Não foram encontrados na literatura dados que justifiquem o tratamento rotineiro da ectopia; 2) O tratamento pode ser utilizado para tratar sintomas associados à ectopia, porém mais sintomas são atribuídos à ectopia do que se pôde confirmar em um estudo controlado; 3) Seriam necessários novos estudos para testar a hipótese de proteção contra o câncer de colo proporcionada pelo tratamento.

## INTRODUCTION

Uterine cervical ectopy is the occurrence of single-layered secreting columnar epithelium (which usually covers the cervical canal, i.e. the endocervix), beyond the external cervical orifice. Thus, the multilayered squamous epithelium typically found in the vagina and exocervix are replaced.^1–3^ This condition has many designations in medical terminology: ectropion, erythroplakia, macula rubra and erosion.^1,2,4,5^

Not all factors involved in the pathogenesis of cervical ectopy are known but there is an association with the action of estrogen.^2,3,6^ Ectopy is rare beyond the menopause and frequent at reproductive ages. It has higher prevalence during pregnancy^2^ and also among users of estrogen-based contraceptives.^6–11^ The rare examinations on newborns that have been reported show high prevalence, probably secondary to estrogens of pregnancy.^12^ There is also, starting in puberty, a negative association with age, even before the menopause. Some studies demonstrated a negative association with the number of years of sexual activity and number of partners.^2,7^

The natural history of ectopy is well established. After its development, a process of metaplasia occurs in the columnar epithelium, known as squamous metaplasia.^1,3^ All women go through this process, which may take months or years, and the exposed columnar epithelium is partially or fully converted into stratified squamous epithelium. The resulting area is known as the transformation zone.^1,3^

The prevalence reported for ectopy ranges from 17 to 50%.^13,14^ Given that its course is usually time-limited, the prevalence estimates in a population will detect only the women with ectopy at that time. In such populations, some women will already have had this condition and others will develop it. It is likely that most women, if not all, will have ectopy at some point during their lifetimes.^4,6^

Cervical ectopy and the associated squamous metaplasia are now considered to be physiological phenomena.^1,15^ Nonetheless, its management has historically consisted of interventions with the purpose of inducing or accelerating its regression. There seems to be a current trend towards less intervention, but it is still very common. Although the spontaneous process of metaplasia almost always leads to reduction or elimination of ectopy, this is a much slower process than the process resulting from treatment. The treatments currently available are electrocoagulation, cryocauterization, laser cauterization and drug treatment.^16^ Cauterization, in its several variants, is the treatment most often used. Patients are treated on an outpatient basis. The efficacy for cauterization is around 90%.^16^

There are several lines of argument that would support routine treatment for ectopy. The most common ones are:

- Protection against cervical cancer. This is probably the argument most generally seen. There is a relationship between squamous metaplasia and induction of squamous cell carcinoma of the cervix.^2,3,15^ Cells undergoing metaplasia are more susceptible to carcinogens. Precancerous lesions often develop at the squamous-columnar junction, i.e. the area of transition between glandular and stratified epithelium, which is the location where metaplasia is most intense.^15^ Thus, theoretically, if this process could be made to occur over a shorter time span and if, by reducing ectopy, metaplasia would be less extensive, there would be a lower risk of cancer.
- Some sexually transmitted microorganisms such as *Chlamydia trachomatis* and *Neisseria gonorrhoeae* preferentially infect glandular epithelium. Ectopy would, by exposing this epithelium, favor infection.^15,17^
- Ectopy consists of secreting epithelium, and it is thus associated with increased mucus production,^2,18^ which may cause discomfort to women. Other symptoms are also sometimes attributed to ectopy, like pelvic pain and postcoital bleeding.^14^

We believe this is a major issue because of the high prevalence of ectopy. If it were decided to treat all women with ectopy, this would entail the utilization of substantial physical and human resources, even though the treatment is not complex. Hence the need to evaluate whether intervention produces any real benefit.

The objective of this study was to assess, through a comprehensive review of the literature, what the alleged indications for and benefits from treating ectopy are and, above all, whether these benefits are purely theoretical or are based on evidence from clinical and/or epidemiological studies.

## METHODS

This study consisted of a literature review including searches in the Medical Literature Analysis and Retrieval System Online (Medline), Literatura Latino-Americana e do Caribe em Ciências da Saúde (Lilacs) and Cochrane Library databases up to July 2006; the Excerpta Medica Database (Embase) database from 1994 to 1999; and specialized books and references in books and selected articles. In addition, two professors of Gynecology at two different public universities in São Paulo, whose work has been focused on conditions of the lower genital tract and colposcopy, were consulted to ascertain whether they knew of any evidence in the literature regarding the benefits from treatment.

The review of the databases included the following approach: any study with ectopy as a main or secondary subject was searched. Once found, the titles and abstracts of the studies were evaluated. When there was any chance that a study somehow addressed the issue of benefits or indications for treatment, even indirectly, it was selected for analysis of its full text.

The protocol for this study was approved by the Ethics Committee of Hospital das Clínicas, Faculdade de Medicina da Universidade de São Paulo (HCFMUSP).

## RESULTS FROM THE LITERATURE REVIEW

### Specialized books

Cartier and Cartier^1^ stated that ectopy is a physiological phenomenon and thus should not be treated. They also argued that, with treatment, the squamous-columnar junction is very often displaced up to the cervical canal, thereby making cervical cancer prevention more difficult. The book does not contain bibliographical references. De Palo^4^ recommended treatment because “stratified epithelium is more physiological in the exocervix”. His opinion was not supported by bibliographical references. Pereyra and Guerra^16^ recommended treatment, but they did not offer any arguments or references to support this.

Singer and Monaghan^3^, Berek et al.^15^, Piato^19^ and Pereyra et al.^20^ did not address the issue of treatment.

### Selected studies

Table 1 shows the results from the database searches. In summary, 2,917 articles were found. Most of these studies addressed the efficacy of treatment approaches promoting ectopy regression, and sometimes the physiological or histological features of this condition as well. Studies dealing with the question of the benefits from routine treatment are rarely seen (Table 2). These studies are presented here, as well as a larger number of other studies showing possible associations between ectopy and certain diseases or symptoms, which could indirectly imply that there are occasional benefits from treatment.

**Table 1.** Distribution of the articles selected, according to total number of references, keywords used and search database

Database searched	Keywords used	Total references	Total selected articles
Medline	NLM ‘Cervix diseases (MeSH) erosion’	423	9
	NLM ‘Cervix erosion (MeSH)’	381	2
	NLM ‘Cervix erosion’	94	3
	NLM ‘(Pubmed) ectopy’	71	17
	NLM ‘Cervix diseases (MeSH) ectopy’	19	1
Bireme	‘Diseases of uterine cervix’	1541	12
	‘Cervix erosion’	6	0
Embase	‘Uterine cervix erosion’	11	1
	‘Uterine cervix disease’	128	0
Lilacs	Ectropion	2	0
	Ectopy	4	0
	Cauterization	11	1
	Diseases of uterine cervix	198	0
Cochrane Library (in controlled trials):	‘Ectopy and cervical’	4	1
	‘Ectropion and cervical’	5	0
	‘Erosion and cervical’	19	0
**Total**		**2917**	**47**

Medline = Medical Literature Analysis and Retrieval System Online; Bireme = Biblioteca Regional de Medicina; Embase = Excerpta Medica Database; Lilacs = Literatura Latino-Americana e do Caribe em Ciências da Saúde.

**Table 2.** Authors who made suggestions regarding treatment for ectopy

Author	Type of work	Year of publication	Recommended treatment	Argument for treatment
Leppaluoto^21^	Opinion article	1974	Yes	Prevention of cervical cancer
Donahue^22^	Opinion article	1976	No	-
Kauraniemi et al.^59^	Cross-sectional study	1978	Yes	Prevention of cervical cancer
De Punzio et al.^61^	Cross-sectional study	1984	No	-
Vonka et al.^60^	Cross-sectional study	1984	Yes	Prevention of cervical cancer
La Vecchia et al.^62^	Case-control study	1985	No	-
Moreira et al.^24^	Opinion article	1990	No	-
Cartier and Cartier^1^	Text in book	1994	No	-
De Luca Brunori et al.^25^	Laboratory study	1994	Yes	Better immunity
Pereyra and Guerra^16^	Text in book	1994	Yes	None
De Palo^4^	Text in book	1996	Yes	Not clear
Madej et al.^23^	Opinion article	1999	No	-
Rocha-Zavaleta et al.^46^	Cross-sectional study	2004	Yes	Prevention of cervical cancer

A set of 48 studies was selected to be presented. One of these was a reference in another study. The two professors consulted said that they were unaware of any clinical evidence in the literature regarding benefits from treatment. No study regarded as relevant was left out of the analysis due to any language difficulty. Copies of eight studies not available in Brazil were imported.

Among the 48 studies selected, four expressed the authors’ opinions, one was a laboratory study and 43 were clinical studies. The clinical studies included the following topics: associations between ectopy and cervical infection due to *Chlamydia trachomatis* and *Neisseria gonorrhoeae*, cytomegalovirus (CMV), human immunodeficiency virus (HIV), human papillomavirus (HPV) and cervical intraepithelial neoplasia (CIN); symptoms of ectopy; and cervical cancer and protection against this cancer by cauterization. Out of these 48 studies, four are not discussed here because they present major methodological flaws.

### Authors’ opinions

Leppaluoto^21^ was in favor of routine treatment, in order to prevent cervical cancer.

Donahue^22^ and Madej et al.^23^ believed that ectopy was a physiological phenomenon and should only be treated when symptomatic. Moreira et al.^24^, in addition to these arguments, maintained that treatment did not prevent cervical cancer.

### Laboratory study

De Luca Brunori et al.^25^ studied asymptomatic women with ectopy and obtained biopsy samples from areas of ectopy and stratified epithelium. They found lower cellular immune activity in areas of ectopy. Based on this finding, they proposed that treatment should be undertaken routinely.

### Clinical studies

#### Chlamydia and gonococcus

Nine cross-sectional studies^7,8,26–32^ using univariate analysis reported an association between cervical *Chlamydia* infection and ectopy. In another four cross-sectional studies^10,11,33,34^ and in one clinical trial,^35^ this association was maintained in multivariate analyses. A further two cross-sectional studies^36,37^ showed that this association disappeared in the multivariate analysis and a cohort study^38^ found a strong tendency towards an association but did not reach significance.

Seven cross-sectional studies^8,11,14,29,30,33,34^ reported that there was no association between gonococcus and ectopy (Table 3).

**Table 3.** List of studies selected according to the association between *Chlamydia* infection and ectopy

Author	Year	Location	Study design	Type of analysis	Association	MA	95% CI	p-value
Ripa et al.^26^	1978	Sweden	Cross-sectional	Univariate	Yes			
Hobson et al.^7^	1980	England	Cross-sectional	Univariate	Yes*	*χ*2 = 9.98		0.01
Tait et al.^27^	1980	England	Cross-sectional	Univariate	Yes	*χ*2 = 12.5		0.0004
Arya et al.^8^	1981	England	Cross-sectional	Univariate	Yes	*χ*2 = 24.34		0.001
Mallinson et al.^32^	1982	England	Cross-sectional	Univariate	Yes*	*χ*2 = 3.96		0.05
Chacko and Lovchik^29^	1984	US	Cross-sectional	Univariate	Yes			
Wood et al.^28^	1984	England	Cross-sectional	Univariate	Yes	*χ*2 = 9.24		0.001
Harrison et al.^10^	1985	US	Cross-sectional	Multivariate	Yes†			
Handsfield et al.^36^	1986	US	Cross-sectional	Multivariate	No			
Blythe et al.^33^	1988	US	Cross-sectional	Multivariate	Yes			
Louv et al.^30^	1989	US	Cross-sectional	Univariate	Yes	RR = 1.49	1.12-1.97	0.005
Paavonen et al.^35^	1989	Sweden	Clinical trial	Multivariate	Yes			
Rahm et al.^31^	1990	Sweden	Cross-sectional	Univariate	Yes	*χ*2 = 9.6		0.01
Rahm et al.^38^	1991	Sweden	Cohort	Univariate	No	RR = 1.78	0.95-3.33	
Stergachis et al.^34^	1993	US	Cross-sectional	Multivariate	Yes	OR = 3.7	2-6.9	
Critchlow et al.^11^	1995	US	Cross-sectional	Multivariate	Yes	OR = 2.4	1.5-3.9	
Jacobson et al.^37^	2000	US	Cross-sectional	Multivariate	No	OR = 1.94	0.40-2.39	

* Association between infection intensity and ectopy;

† Multivariate analysis did not include ectopy individually but, rather, a score including ectopy. MA = measurement of association; CI = confidence interval; χ2 = chi-squared; OR = odds ratio; RR = relative risk; US = United States.

#### Cytomegalovirus

Collier et al.^39^ studied the relationship between sexual activity and CMV infection. He found an association between cervical CMV infection and ectopy that disappeared in multivariate analysis that included age, schooling level and race.

#### HIV

Moss et al.^40^ conducted a cross-sectional study among 70 couples in which the men were HIV-positive. They found that the women with ectopy were at greater risk of being HIV-positive, and that this risk was maintained in multivariate analysis: odds ratio (OR) = 5; 95% confidence interval (CI) = 1.7-14.7; p = 0.007.

In a cross-sectional study on 97 HIV-positive women, Clemetson et al.^18^ found a higher frequency of HIV isolation from the cervix and vagina in the women with ectopy. This association remained in the multivariate analysis: OR = 5; 95% CI = 1.5-16.9; p = 0.006.

Plourde et al.^41^ conducted a cohort study on 134 HIV-negative women with genital ulcers. The group was followed up monthly for six months and infection with the HIV virus during this period was correlated with the women's characteristics. They found an association between ectopy and risk of HIV infection: relative risk (RR) = 3.9; 95% CI = 1.2 – 12.7; and also an association between ectopy and shorter time for seroconversion.

Mati et al.^42^ conducted a cross-sectional study comprising 4,404 women in family planning clinics, to assess the association between the risk of HIV infection and contraceptive methods. Ectopy was also assessed, given the association with oral contraceptive use. Out of all of these women, 4.9% were HIV-positive. No association was found between HIV infection and ectopy.

Moscicki et al.^43^ conducted a cross-sectional study among 189 HIV-positive adolescents and 92 HIV-negative adolescents. Factors associated with their HIV status, including ectopy, were studied. Ectopy was measured through computerized analysis. The univariate analysis showed a negative association between ectopy and HIV infection, which remained in the multivariate analysis: OR = 0.55; 95% CI = 0.31-0.98; p = 0.04 (Table 4).

**Table 4.** List of studies selected according to association between ectopy and HIV infection

Author	Year	Location	Study design	Type of analysis	Association	Measurement of association	95% CI	p-value
Moss et al.^40^	1991	Kenya	Cross-sectional	Multivariate	Yes	OR = 5	1.7-14.7	0.007
Clemetson et al.^18^	1993	Kenya	Cross-sectional	Multivariate	Yes*	OR = 5	1.5-16.9	0.06
Plourde et al.^41^	1994	Kenya	Cohort	Univariate	Yes	RR = 4.9	1.5-15.9	0.02
Mati et al.^42^	1995	Kenya	Cross-sectional	Univariate	No	OR = 1.3	0.7-2.1	–
Moscicki et al.^43^	2001	US	Cross-sectional	Multivariate	Yes (negative)	OR = 0.55	0.31-0.98	0.04

* Association between ectopy and viral isolation from vaginal and cervical discharges among HIV-positive women.

CI = confidence interval; OR = odds ratio; RR = relative risk; US = United States.

#### HPV and CIN

Toon et al.^44^ conducted a cross-sectional study among 210 women to identify factors associated with inflammatory cytological conditions. He found a much higher frequency of HPV in biopsies from women with ectopy (all the participants underwent biopsy; statistical data not available).

Duttagupta et al.^45^ conducted a cross-sectional study on 850 women to assess the validity of detection of HPV subtypes 16 and 18 (which were considered to be oncogenic) as an approach for cervical cancer screening. An association between ectopy and HPV (OR = 2.09; p = 0.005) was found.

In a cross-sectional study, Rocha-Zavaleta et al.^46^ found a higher general HPV rate and also a higher rate of HPV 16 in women with ectopy. However, the association was not significant, either for HPV in general (OR = 2.06; 95% CI = 0.99-4.33) or for HPV 16 (OR = 6.47; 95% CI = 0.88-133). They proposed routine treatment for ectopy, in areas with high HPV prevalence, to prevent cervical cancer.

Castle et al.^47^ studied the relationship between ectopy and two different groups of HPV: the alpha 9 group (mostly oncogenic) and the alpha 3/alpha 15 group (mostly non-oncogenic). A significantly higher rate of ectopy was found in women with the non-oncogenic group than in the other group (control group). They also found an association between infection in the oncogenic group and younger age. They suggested that the greater oncogenicity in this group could be due to higher affinity to metaplastic epithelium in young women.

Moscicki et al.^48^ conducted a case-control study on 18 adolescents with CIN and 204 controls. Among other variables, ectopy was assessed using computer-processed images. They found an association between these variables (RR = 4.27; 95% CI = 1.45-12.45).

Sarkar and Steele^49^ conducted a study on 100 women who had been referred to a clinic for ectopy treatment. All of these women had normal cervical cytology. All underwent colposcopy and, if needed, cervical biopsy prior to treatment. CIN was found in 19 patients, and five of them had high-grade lesions. The authors considered that this CIN prevalence (both the high and low-grade, types) was greater than the expected rate for this population.

Moscicki et al.^50^ conducted a case-control study comprising 75 adolescents with CIN and 75 controls. These were followed up for an average of 32 months. The authors found an association between CIN and the intensity of metaplasia (which is related to ectopy), immediately before CIN was diagnosed: OR = 3; 95% CI = 1.3-6.82.

#### Symptoms

Goldacre et al.^14^ conducted a cross-sectional study correlating epidemiological features and symptoms with ectopy. They studied 1,498 women who had sought out a family planning center. Ectopy was evaluated through clinical examination. The women were asked about symptoms that are attributed to ectopy, such as vaginal discharge, vulvar pruritus, low back pain, postcoital bleeding, painful intercourse, dysuria, nocturia, and pollakiuria. Tests for pathogenic microorganisms of the cervix and vagina were also conducted: *Trichomonas vaginalis*, fungi, and gonococcus and the bacterial flora of the vagina. The person who performed the examination (for detecting ectopy) was unaware of the subjects’ symptoms and the person who evaluated the symptoms was unaware of the existence of ectopy. Ectopy was found in 550 women (36.7%). There was no association between ectopy and fungal, *Trichomonas vaginalis* or gonococcal infection. No differences were seen in the bacterial flora of the vagina. In the multivariate analysis, which included mucus discharge, nocturia, parity, contraceptive method and evaluating physician, only the associations with mucus discharge and nocturia remained (p < 0.05).

#### Cervical cancer (Table 5)

**Table 5.** Studies that evaluated the association between ectopy and cervical cancer

Author	Type of Study	Year of publication	Association
Jiangxi Group^52^	Case-control	1986	Yes
Murthy et al.^55^	Cohort	1990	No
Zhang and Xu^53^	Case-control	1990	Yes
Juneja et al.^54^	Case-control	1993	Yes

Simm and Doltaniak^51^ suggested that there was an association between ectopy and cervical cancer. However, their conclusion was based on a study with serious methodological flaws and therefore it will not be discussed further here.

The Jiangxi Cooperative Group of Cervical Cancer^52^ conducted a case-control study in China, in 1980. They studied 306 women with cervical cancer and 306 controls. Thirty-six variables were investigated through direct interview. They found an association with ectopy.

Zhang and Xu^53^ conducted a case-control study comprising 125 cases of cervical cancer and 125 controls in China. Associations between 39 variables and cancer occurrence were assessed. In the multivariate analysis, the association with ectopy was maintained, among others.

Juneja et al.^54^ conducted a cross-sectional study to identify variables that were associated with cervical cancer. They studied 67,000 women who underwent a cytology test. At the time of data collection, all women underwent an examination. The cervix was classified as normal or presenting ectopic bleeding upon touch, “suspicious appearance” or “unhealthy” appearance. A total of 250 women (0.4%) had cytological findings suggestive of invasive cancer, which, for the purposes of that study, was considered to be the definition of cancer. A significant association between cancer and all variables was seen, including ectopic bleeding upon touch.

Murthy et al.^55^ organized a cohort consisting of 1,107 women with cytological findings suggestive of CIN. They were followed up at three to six-month intervals with colposcopy, and biopsy if necessary, along with cytological tests. Associations between progress to *in situ* carcinoma and the following variables were studied: age (35 years or over), use or nonuse of contraception, parity, fetal losses, Herpes simplex I and II status and ectopy. Over the course of 78 months of follow-up, 75 women progressed to *in situ* carcinoma. In the multivariate analysis, only the association with age at marriage was maintained (p = 0.02). No association with ectopy was found.

#### Cancer prevention using cauterization

Kanka et al.^56^, Bouda and Dohnal^57^ and Peyton et al.^58^ conducted studies to investigate cancer prevention using cauterization. However, these studies presented methodological flaws that made them inconsistent, and thus they will not be discussed further here.

Kauraniemi et al.^59^ conducted a cross-sectional population-based study among 429,832 women who underwent cervical cancer screening with cytological tests. They correlated histories of cauterization due to any indication at any time with the detection of malignant or premalignant histological lesions. They found a negative association between cauterization and neoplastic and preneoplastic cervical lesions. The relative risks were: low-grade dysplasia, 0.40; high-grade dysplasia, 0.24; *in situ* carcinoma, 0.23; and invasive carcinoma, 0.15. After stratification by age, the association remained. After stratification by marital status, the association disappeared for single women. They concluded that cauterization protected against cervical cancer and noted that this protection might be greater than the protection resulting from mass screening programs.

Vonka et al.^60^ conducted a cross-section study among 10,683 women to identify risk factors for CIN. They found a protective effect for history of cauterization: 24.6% of the controls had cauterization versus 13.4% of the women with cervical intraepithelial neoplasia grade I (CIN I), 6.9% of those with CIN II, 8.7% of those with CIN III and 9.5% of those with *in situ* carcinoma (p < 0.05). They concluded that ectopy should be cauterized to prevent cervical cancer.

De Punzio et al.^61^ conducted a cross-sectional study among 2,001 women in a private clinic for cervical cancer prevention. They compared the prevalence of preneoplastic and neoplastic lesions with histories of cauterization at any time and due to any indication. They found no association.

La Vecchia et al.^62^ conducted a case-control study with the specific objective of assessing whether electrocoagulation of ectopy protected against cervical cancer. Two case-control studies were conducted simultaneously. In the first study, the cases were 191 women with invasive cervical cancer and there were 191 controls. The second study had the same format as the first one, except that the cases were women with CIN.

In their first study, on invasive carcinoma, the univariate analysis showed an apparent protective effect among cauterized women: RR = 0.42; 95% CI = 0.22-0.82. After adjusting for the number of cervical cytological tests (none, one, or two or more), it was found that the RR increased to 0.83 and was no longer significant (95% CI = 0.40-1.72). In the multivariate analysis, which included age, education, parity, number of sexual partners, age at first sexual intercourse and use of oral contraceptives, it reached as high as 0.94. Within the same stratum of cytological tests, adjusting for cauterization did not change the relative risk. In the second study, among women with CIN, the same sequence of results was found.

These authors (La Vecchia et al.^62^) cited the aforementioned studies of Peyton et al.^58^, Kauraniemi et al.^59^ and Vonka et al.^60^ as major references on this issue. They stated that, differently from those other studies, their study showed evidence against the protective effect of cauterization for cervical cancer (Table 6).

**Table 6.** List of studies selected according to the association between cervical cancer and cauterization of ectopy

Author	Year	Location	Type of analysis	Protection	Measurement of protection*
Kauraniemi et al.^59^	1978	Finland	Univariate	Yes	RR = 0.15
Vonka et al.^60^	1984	Czechoslovakia	Univariate	Yes	
De Punzio et al.^61^	1984	Italy	Univariate	No	
La Vecchia et al.^62^	1985	Italy	Multivariate	No	RR = 0.94

* Confidence intervals and p-values not made available by the authors. RR = relative risk.

### DISCUSSION OF THE STUDIES

As mentioned earlier, few studies have been conducted to evaluate the benefits from routine treatment for ectopy. In the Cochrane Library's review, for example, only the efficacy of treatments for ectopy regression was discussed.

The small number of studies reviewing the validity of routine treatment indicates that the medical scientific community has not been greatly interested in answering this question, even though treatment for ectopy is a very common intervention. The large number of articles dealing exclusively with treatment approaches corroborates this statement.

The lack of correlation between scientific evidence for benefits and the widely practiced treatments makes it clear that, in the medical field, the clinical management methods are not only supported by recent scientific knowledge. Some authors have pointed out that Medicine has the characteristics of a practice that is supported by scientific knowledge but connected to other areas of social life^63,64^ and based on the physician's performance. The physician is not only a knowledge holder but also an individual who possesses values, beliefs and motivations.^65^

If, on the one hand, up-to-date scientific knowledge is paramount, as seen in the present study, physicians tend to recognize knowledge acquired both at medical school, during interactions with teachers, and in their clinical experience, in autonomous practice. Physicians make therapeutic decisions based on a set of sources, with varying levels of patient involvement.

Thus, there were several articles that, although they did not address the main concern of the present study, suggested associations between ectopy and certain diseases or symptoms. These will be discussed now.

#### Gonococcus

None of the seven studies investigating associations between ectopy and gonococcal infection was able to confirm such an association.^8,11,14,29,30,33,36^ It can be concluded these conditions are not associated.

#### Chlamydia

An association between *Chlamydia* infection and ectopy can be assumed to be very likely. Out of the 17 studies investigating this, 14 found an association (five also in multivariate analysis) and only three did not show it. Among these three, the study by Rahm et al.^38^ showed a higher frequency of infection among women with ectopy, but had a small sample and was not statistically significant (OR = 1.78; 95% CI = 0.95-3.33).

It can also be assumed that a cause-effect relationship is likely, such that ectopy favors infection, given the affinity of *Chlamydia* for glandular epithelium.^17^ Supposing these hypotheses to be true, it can be assumed that treatment for ectopy could reduce the risk of *Chlamydia* infection.

There would be no sense, however, in treating all women with ectopy with this purpose. Based on the *Chlamydia* studies reviewed here, the infection prevalence ranges from 3.7% to 35%; the former rate is probably closer to that of the general population, since it was estimated in a primary care service.^11^ In Brazilian studies, the infection rates have ranged from 4% to 11.2%.^66–68^ It should be underlined that most infected women are asymptomatic, and represent a problem only in that they may be possible carriers of infection.^17^ Since the prevalence of ectopy is high, its treatment would be an intervention of little benefit, given the large population to be treated. Moreover, there are strategies for managing *Chlamydia* infection in the general population that are more effective. Nonetheless, there could be specific situations in which such interventions would be effective, for instance, in cases of women with high exposure to sexually transmitted diseases and some difficulty in getting their partners to use condoms regularly.

#### HIV

A similar discussion holds for HIV infection. It can be assumed that women with ectopy are likely to be susceptible to HIV infection. However, the studies presented here showed contradictory results. One likely explanation for these inconsistencies is that, when exposed to HIV, women with ectopy are at higher risk of infection, probably due to lower immune competence of their glandular epithelium.^24^ Ectopy is, however, inversely associated with age and sexual exposure.^11^ Women with ectopy would thus comprise a group at lower risk of HIV exposure, since they are on average younger and have lower sexual exposure. In the study by Moss et al.^40^, all the women were exposed to an HIV-positive partner and, consequently, women with ectopy were more infected. In the studies by Mati et al.^42^ and Moscicki et al.^43^, the women with ectopy were drawn from the general population and thus were less likely to be exposed to HIV, hence the lack of association or negative association found. In the study by Plourde et al.^41^, since all the women had genital ulcers, they probably comprised a group with higher exposure to sexually transmitted diseases and therefore to HIV.

On the other hand, assuming that this population has higher susceptibility, it would be pointless to treat all women with ectopy to reduce this risk, even with regard to fatal conditions. It would be an extensive therapeutic intervention of low efficacy. Even if it were believed that treatment provided some protection, it would never provide full protection. In the same way as with *Chlamydia* infection, particular situations could be envisaged in which treatment would be justifiable, for example, cases of HIV-negative women with HIV-positive partners.

The alleged benefits in these specific situations are, however, only theoretically inferred on the basis of the present review. They have not been proven across the population. For example, among the studies reviewed that dealt with the issues of HIV and *Chlamydia*, none of them even mentioned treatment for ectopy. Their focus was basically on identifying risk markers for those infections.

#### Symptomatic ectopy

It is accepted that ectopy should be treated when there are symptoms attributable to this condition that cause discomfort. However, Goldacre et al.^14^ showed that more symptoms are attributed to ectopy than are actually caused by it.

#### HPV and CIN

All four studies dealing with HPV infection and ectopy^44,45,59,60^ showed an association between these conditions. Three of them showed associations with oncogenic subtypes. Three studies dealing with associations between ectopy and CIN were reviewed.^48,50^ The study by Sarkar and Steele^49^ suggested that there was an association between CIN and ectopy, and both studies by Moscicki et al.^48,50^ showed an association between these conditions. If it is assumed that there is an association between ectopy and both HPV and CIN, and that ectopy favors the occurrence of these two conditions (a more likely scenario) rather than these conditions favoring the occurrence of ectopy, ectopy should be taken to be a risk factor for cervical cancer.

However, the seven studies discussed above do not provide evidence for such an association. CIN and HPV do not have a direct relationship with cervical cancer, even when oncogenic virus subtypes are involved. Based on these studies, it can be said that the ultimate argument in support of carrying out interventions to treat ectopy would be if it prevented cervical cancer. This issue will be further discussed below.

#### Cervical cancer

The existence of an association between ectopy and cervical cancer would, in theory, be the most important argument in favor of routine treatment. Cervical cancer is a severe disease that is usually fatal when not treated in a timely manner.^2^ An intervention leading to a reduction of, for example, 15% of the incidence rate expected for a specific population (taking the protection factor estimated in the study by Kauraniemi et al.,^59^) would have a great impact on the mortality and morbidity caused by this disease. If this protection actually exists, it would justify searching for and treating all detected cases of ectopy.

Both of the case-control studies that investigated this issue (Jiangxi Co-operative Group of Cervical Cancer^52^ and Zhang and Xu^53^) reported that such an association existed. In both studies, however, the alleged risk factors were assessed through a questionnaire applied to women and it is possible that there may have been some classification bias. When cancer patients are clinically examined before their cancer has been diagnosed, they could be considered to present ectopy (erosion), which can be clinically mistaken for incipient cancer.

In the study by Juneja et al.^54^, bias is even more likely. The cancer diagnosis was made based on cytological data collected during the same evaluation as when ectopy was detected. In this case, it is very likely that well-established cancer was misdiagnosed as “ectopic bleeding upon touch”.

In contrast, in the cohort studied by Murthy et al.^55^, all the participants underwent colposcopy, which is the best approach for diagnosing ectopy. These authors did not find any association between ectopy and progression to *in situ* carcinoma.

#### Cancer prevention using cauterization

The studies by Kauraniemi et al.^59^ and Vonka et al.^60^ suggested that cauterization would have a protective effect, but not all possible confounders were properly controlled for.

The most convincing study is certainly the one by La Vecchia et al.^62^ Based on arguments developed in previous studies, they questioned them using proper methodology. Although three earlier studies had demonstrated apparent protection, the study by La Vecchia et al.^62^ was specifically designed to explore this issue and showed that treatment for ectopy, is a confounder for cervical cytological findings. The protection factor, which was of magnitude similar to what had been found by other authors, disappeared after controlling for the number of cytological tests. After stratifying according to the number of cytological tests, the history of cauterization did not change the risk of cervical cancer.

Therefore, it can be said that the current evidence does not support the hypothesis that treatment for ectopy provides protection against cervical cancer. It is remarkable that the study by La Vecchia et al.^62^ was published as long ago as 1985. In the present review, no studies of more recent date that retested this hypothesis were found.

## CONCLUSION

The present study allows the following conclusions:

- No data in the medical literature was found supporting routine treatment for ectopy.
- Treatment can be used to relieve occasional symptoms associated with ectopy. However, more symptoms are attributed to this condition than can be confirmed in a controlled study.
- Further studies designed to test the hypothesis that protection against cervical cancer is provided by treatment for ectopy are needed.
